# Chronic hyperinsulinemia promotes human hepatocyte senescence

**DOI:** 10.1016/j.molmet.2022.101558

**Published:** 2022-07-21

**Authors:** Ritesh K. Baboota, Rosa Spinelli, Malin C. Erlandsson, Bruna B. Brandao, Marsel Lino, Hong Yang, Adil Mardinoglu, Maria I. Bokarewa, Jeremie Boucher, C. Ronald Kahn, Ulf Smith

**Affiliations:** 1 Lundberg Laboratory for Diabetes Research, Department of Molecular and Clinical Medicine, Sahlgrenska Academy, University of Gothenburg, Gothenburg, Sweden; 2 Department of Translational Medical Sciences, Federico II University of Naples, Naples, Italy; 3 URT Genomics of Diabetes, Institute of Experimental Endocrinology and Oncology, National Research Council, Naples, Italy; 4 Department of Rheumatology and Inflammation Research, Institute of Medicine, University of Gothenburg, Gothenburg, Sweden; 5 Rheumatology Clinic, Sahlgrenska University Hospital, Gothenburg, Sweden; 6 Joslin Diabetes Center and Harvard Medical School, One Joslin Place, Boston, MA, USA; 7 Science for Life Laboratory, KTH—Royal Institute of Technology, Stockholm, Sweden; 8 Metabolic Disease, Evotec International GmbH, Göttingen, Germany

**Keywords:** Hyperinsulinemia, Senescence, Hepatocytes, NAFLD, Dasatinib, Quercetin

## Abstract

**Objective:**

Cellular senescence, an irreversible proliferative cell arrest, is caused by excessive intracellular or extracellular stress/damage. Increased senescent cells have been identified in multiple tissues in different metabolic and other aging-related diseases. Recently, several human and mouse studies emphasized the involvement of senescence in development and progression of NAFLD. Hyperinsulinemia, seen in obesity, metabolic syndrome, and other conditions of insulin resistance, has been linked to senescence in adipocytes and neurons. Here, we investigate the possible direct role of chronic hyperinsulinemia in the development of senescence in human hepatocytes.

**Methods:**

Using fluorescence microscopy, immunoblotting, and gene expression, we tested senescence markers in human hepatocytes subjected to chronic hyperinsulinemia *in vitro* and validated the data *in vivo* by using liver-specific insulin receptor knockout (LIRKO) mice. The consequences of hyperinsulinemia were also studied in senescent hepatocytes following doxorubicin as a model of stress-induced senescence. Furthermore, the effects of senolytic agents in insulin- and doxorubicin-treated cells were analyzed.

**Results:**

Results showed that exposing the hepatocytes to prolonged hyperinsulinemia promotes the onset of senescence by increasing the expression of p53 and p21. It also further enhanced the senescent phenotype in already senescent hepatocytes. Addition of insulin signaling pathway inhibitors prevented the increase in cell senescence, supporting the direct contribution of insulin. Furthermore, LIRKO mice, in which insulin signaling in the liver is abolished due to deletion of the insulin receptor gene, showed no differences in senescence compared to their wild-type counterparts despite having marked hyperinsulinemia indicating these are receptor-mediated effects. In contrast, the persistent hyperinsulinemia in LIRKO mice enhanced senescence in white adipose tissue. *In vitro*, senolytic agents dasatinib and quercetin reduced the prosenescent effects of hyperinsulinemia in hepatocytes.

**Conclusion:**

Our findings demonstrate a direct link between chronic hyperinsulinemia and hepatocyte senescence. This effect can be blocked by reducing the levels of insulin receptors or administration of senolytic drugs, such as dasatinib and quercetin.

## Highlights

• Chronic hyperinsulinemia promotes human hepatocyte senescence via the p53/p21 pathway.
• Hyperinsulinemia-induced senescence requires the PI3K/AKT/mTOR signaling pathway.
• Hyperinsulinemia makes human hepatocytes more susceptible to stress-induced senescence.
• Insulin sustains pro-senescence cellular stress by enhancing Cyclin D1 expression.
• Dasatinib and quercetin inhibit the hyperinsulinemia-induced hepatocyte senescence.

## 1 Indtroduction

Non-alcoholic fatty liver disease (NAFLD) has rapidly become a common cause of liver disease worldwide. NAFLD is included in a spectrum of liver diseases with clinical and histological abnormalities ranging from simple steatosis to non-alcoholic steatohepatitis (NASH), which can progress to more severe stages such as cirrhosis and hepatocellular carcinoma (HCC) [1]. The severity of the disease, combined with the fact that there is currently no approved pharmacological treatment, has accelerated efforts to understand its pathogenesis and identify new therapeutic targets.

Recently, therapeutically targeting cellular senescence to prevent or treat multiple age-related diseases, including metabolic disorders, has received considerable attention. Cellular senescence is one of the hallmarks of aging and contributes to age-related dysfunction and disease [2]. Senescent cells are characterized by a stable cell-cycle arrest, secretion of senescence-associated secretory phenotype (SASPs), macromolecular damage and altered metabolism that is triggered by stressful insults and certain genetic and physiological processes [3,4]. Cell cycle arrest in senescence is largely mediated via activation of either one or both p53/p21 and p16/pRB tumor suppressor pathways [4]. Studies in both mice and man have shown that NAFLD is accompanied by an increase in hepatic senescent cells [5,6]. Our recent findings also showed that hepatic senescence is strongly associated with the different histopathological features of the disease and a strong predictor of NAFLD/NASH (our unpublished data). However, a causal role for senescence in the development and progression of NAFLD has yet to be established in man but data from mouse studies have shown that eliminating senescent cells alleviates hepatic steatosis [5]. Thus, cellular senescence is a component of many common disorders and has great potential as a target for prevention or treatment of human NAFLD.

Several types of metabolic stress can promote senescence including mitochondrial dysfunction, reactive oxygen species production and hyperglycemia [3,4]. Prospective studies have also shown that NAFLD is strongly associated with metabolic disorders such as obesity and Type 2 diabetes (T2D) suggesting a role of insulin resistance and/or the compensatory hyperinsulinemia which result in defective lipid metabolism and hepatic triglyceride accumulation [7]. It has recently been demonstrated that hyperinsulinemia induces a premature senescent transcriptomic and secretory profile in mature adipocytes [8] and also causes insulin resistance in neurons resulting in a senescence-like phenotype [9]. Furthermore, our recent study documented a strong association between insulin resistance, elevated insulin levels and senescence in the livers of NAFLD/NASH patients (our unpublished data). However, the direct effect of chronic hyperinsulinemia, which usually compensates insulin resistance, on hepatocyte senescence has never been studied prior to the current work.

## 2 Material and methods

### 2.1 Animals

Generation and characterization of LIRKO mice and their littermate controls have been described previously [10]. Both male and female mice (16 weeks old; C57BL/6) were maintained on a 12 h light/dark cycle with ad libitum access to water and food (normal chow diet, 21% fat by calories). All protocols for animal use and euthanasia were reviewed and approved by the Animal Care Committee of the Joslin Diabetes Center and were in accordance with NIH guidelines.

### 2.2 Cell culture and treatment

IHH cells (immortalized human hepatocytes [11]) were kindly provided by Prof. Jan Boren (University of Gothenburg, Sweden). Cells were cultured in Williams E medium (Life Technologies), supplemented with 10% Fetal Bovine Serum (FBS) and 1% penicillin/streptomycin. All experiments were performed between passage 7 and 12.

In order to study the effects of insulin on senescence in hepatocytes, IHH cells were treated with different concentrations of insulin (20 and 100 nM) or IGF1 (100 ng/ml, equivalent to 13 nM) for 6, 24 and 72 h (chronic treatment). The pharmacological mapping of insulin-induced-signaling in IHH cells was performed by pre-incubating cells 1 h before insulin stimulation with culture medium containing the indicated concentrations of a LY294002 (PI3K inhibitor), MK2206 (AKT inhibitor) and Rapamycin (mTOR inhibitor).

As a model of senescence, IHH cells were treated with 2 μM doxorubicin (DOX) for 2 h in Williams’ E medium supplemented as described above. Cells were then washed once and replenished with fresh media, followed by incubation for 72 h. To study the effects of chronic hyperinsulinemia in senescent hepatocytes, DOX-treated cells were incubated with or without insulin (100 nM) for 72 h. Furthermore, to study the effects of the senolytic agents Dasatinib (D) and Quercetin (Q)), DOX-treated cells were incubated with insulin (100 nM) in the presence or absence of the combination of D (0.2 μM) and Q (10 μM).

We used recombinant regular human insulin (Actrapid, Novo Nordisk), recombinant human IGF-I (Life technologies), DOX, D, Q and Rapamycin (Sigma–Aldrich), LY294002 (Calbiochem) and MK2206 (Cayman Chemical).

### 2.3 Immunoblotting

Total proteins from human cells and frozen mouse tissues were extracted in ice-cold lysis buffer (25 mM Tris–HCl, pH 7.4, 0.5 mM EGTA, 25 mM NaCl, 1% (vol/vol) IGEPAL CA-630, 1 mM Na3VO4 and Protease Inhibitor Cocktail (Sigma–Aldrich). Protein content was determined using Pierce BCA Protein Assay kit (Thermo Fisher Scientific). Proteins were separated on 4–12% NuPAGE gels (Thermo Fisher Scientific) and transferred to nitrocellulose membranes (Bio-Rad). The membranes were probed with the following antibodies: p21 (sc-6246, Santa Cruz Biotechnology), p53 (2527, Cell Signaling Technology), Phospho-Histone H2A.X (80312, Cell Signaling Technology), MDM2 (86934, Cell Signaling Technology), CCND1 (55506, Cell Signaling Technology), β-actin (sc-47778, Santa Cruz Biotechnology) and GAPDH (sc-47724, Santa Cruz Biotechnology) and the HRP-conjugated secondary antibodies, anti–rabbit IgG (7074, Cell Signaling Technology) and anti–mouse IgG (7076, Cell Signaling Technology). Proteins were visualized using the ChemiDoc Imaging System (Bio-Rad) and quantification was performed with normalization against loading controls.

### 2.4 Immunocytochemistry

IHH cells grown on coverslips were treated with DOX and Insulin as described above. Cells were fixed in 4% formaldehyde for 15 min and permeabilized in 0.1% Triton X-100 for 5 min. Cells were then blocked with 5% FBS for 1 h followed by incubation with primary antibodies against CCND1, p53, and Phospho-Histone H2A.X for 1 h. After washing in PBS and incubation with secondary antibody conjugated with Alexa-488 (Molecular Probes) for 1 h, nuclei were stained by DAPI and the coverslip was mounted with fluorescence mounting medium (Invitrogen). Pictures were obtained using a Zeiss Axio Observer. Image analysis was performed using an in-house macro in ImageJ (v.1.52 h, NIH) with nuclei being counted, the total area determined, and a static threshold applied to all images for each of the fluorescent channels to determine positively stained area.

### 2.5 Quantitative real-time PCR

Total RNA from human cells and frozen mouse liver tissues was isolated using E.Z.N.A. total RNA kit (Omega Bio-tek). Quantification of RNA was performed with NANODROP 1000 Spectrophotometer, followed by cDNA synthesis using High Capacity cDNA kit according to the manufacturer's recommendations. Gene expression was then analyzed using a Quant Studio 6 Flex TaqMan system (Applied Biosystems). The primers and probes were either designed or ordered commercially as predesigned TaqMan probe kits (Assay On-Demand; Thermo Fisher Scientific). Relative expression was calculated using the ΔΔCt method with normalization to 18 S rRNA. AoDs, probes and primers and endogenous controls are listed in Supplementary Table 1.

### 2.6 Flow cytometry

IHH cells were treated with DOX and insulin as described above. Subsequently, cells were harvested, washed with PBS and resuspended in FACS buffer (PBS with 10% FCS and 0.05% NaN3). Cells were counted using an automated cell counter (Sysmex KX-21). 5 × 10^5^ cells were centrifuged in a V-bottom polypropylene plate, resuspended in 100 μl cytofix/cytoperm (BD Biosciences) and incubated for 60 min. Permeabilised cells were washed once with Perm/wash (BD Biosciences), pelleted and resuspended in 50 μl perm/wash containing 20 μg/ml 7AAD (Invitrogen, Waltham, Massachusetts, USA) and incubated for 60 min. Stained cells were washed 3 times with perm/wash and resuspended in 200 μl FACS buffer. The samples were analyzed with a BD FACSVerse instrument (BD Biosciences) and the data analysis was performed using the FlowJo software (Tree Star).

### 2.7 RNAseq analysis

Hepatic RNA-seq (raw fastq files) of NAFLD cohort included 10 normal samples, 50 patients with NAFL and 155 patients with NASH retrieved from European Nucleotide Archive database (https://www.ebi.ac.uk/ena/) under accession number SRP217231. Kallisto [12] was used for estimating the count and TPM (transcripts/million) values of transcripts based on the Ensembl human reference genome (Version 102, GRCh38.p13). The sum value of the multiple protein-coding transcripts of a gene was used as the expression value of this gene. Differences among groups in gene TPMs were detected by Mann–Whitney U test and p < 0.05 was considered statistically significant.

### 2.8 Statistics

Statistical analysis was performed with GraphPad Prism 9.0 software (GraphPad Software Inc). Results are presented as means ± SEM of at least three independent experiments as indicated in the figure legends. Normal distribution of continuous variables was tested using the Shapiro–Wilk test. Normally distributed data were compared between groups by unpaired Student's t-test (two-tailed). Within-group comparisons between matched samples were performed using paired two-tailed Student t-test or one-way repeated measures ANOVA followed by post hoc test as appropriate. Not normally distributed data were compared between groups by nonparametric tests (two-tailed), as appropriate. The correlation between quantitative variables was tested by Spearman's rank correlation test. p ≤ 0.05 was considered statistically significant.

## 3 Results

### 3.1 Prolonged exposure of human hepatocytes to hyperinsulinemia enhances cell senescence

**Figure 1:**
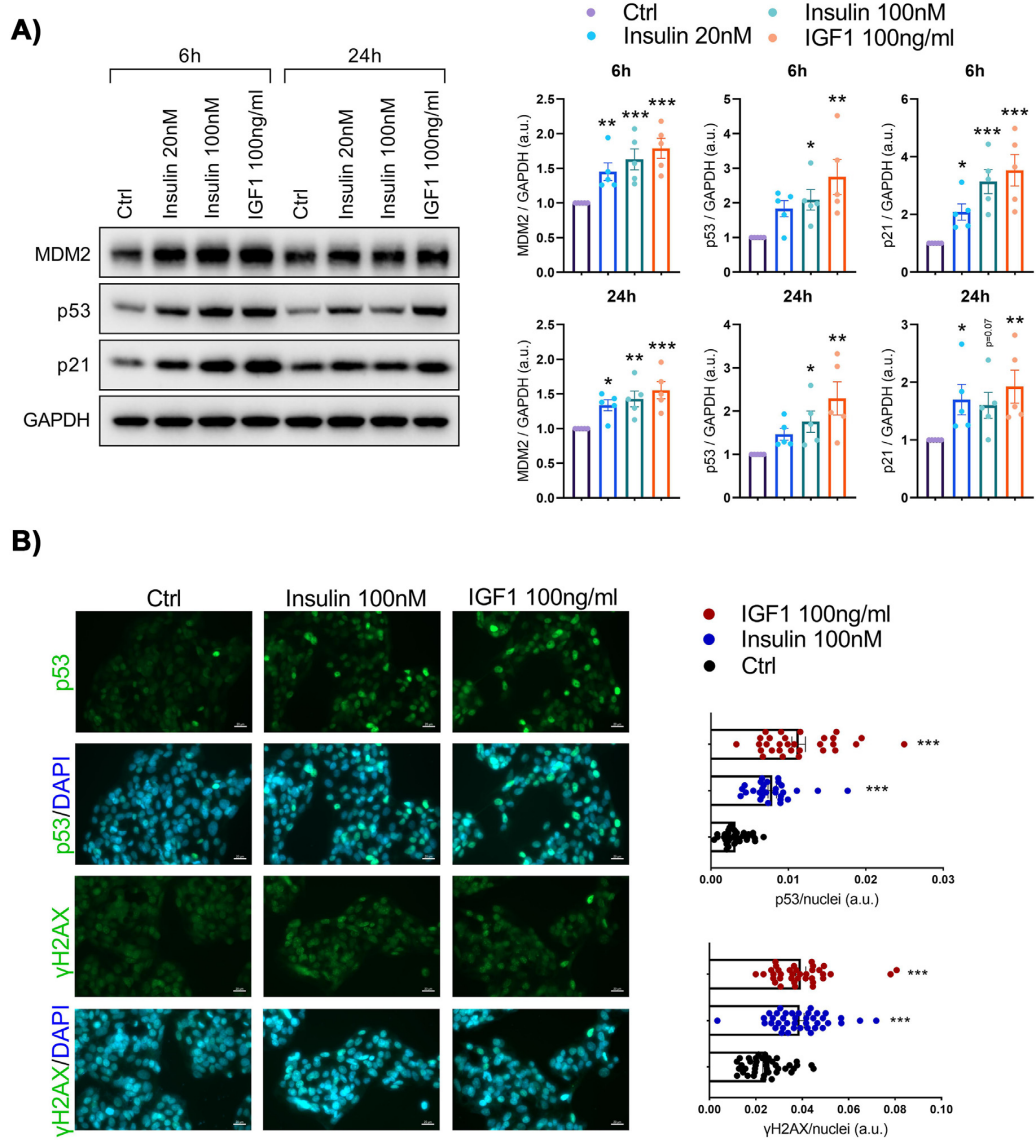
**Senescence markers are increased in human hepatocytes following insulin or insulin-like growth factor 1 (IGF1) exposure**. (A) Representative immunoblots of respective proteins in IHH cells treated with insulin (20 nM and 100 nM) or IGF1 (100 ng/ml) for 6 h and 24 h. Bar graphs showing the relative protein quantification, normalized to GAPDH (n = 5). (B) Representative immunofluorescent images of IHH cells treated with insulin (100 nM) or IGF1 (100 ng/ml) for 24 h, stained for p53 (green), γH2AX (green), and nuclei (DAPI, blue). Scale bar represents 20 μm. Bar graph displays fluorescence intensities quantified using ImageJ and normalized to number of nuclei (n = 3, 8–10 randomly chosen fields from each experiment). Data are shown as mean ± SEM. Dots represent individual level data. Statistical significance was determined using one-way ANOVA followed by Dunnett *post-hoc* test or Kruskal–Wallis with *post-hoc* Dunn's test. ∗p < 0.05, ∗∗p < 0.01, ∗∗∗p < 0.001. ∗*vs.* Ctrl. a.u., arbitrary unit.

To investigate the effect of prolonged hyperinsulinemia on hepatic senescence, we exposed immortalized human hepatocytes (IHH cells) to insulin (20 and 100 nM) for 6, 24 or 72 h. At all-time points, insulin induced a significant increase in the protein senescence markers MDM2, p53, and p21 (Figure 1A; Supplementary Fig. 1a). IGF1, which activates a homologous signaling pathway and can cross-activate with insulin receptor [13], produced a similar increase in senescence markers (Figure 1A; Supplementary Fig. 1 a). The increase in p53 by insulin and IGF1 was also confirmed using immunostaining (Figure 1B). Furthermore, insulin- and IGF1-treated cells exhibited puncta of γH2AX staining signals, which is typical of senescence-associated DNA damage (Figure 1B). Additionally, insulin and IGF1 treatment lead to increase in mRNA levels of *p16* and SASPs markers including *IL18*, *MMP3*, *IL32* and *IL8* (Supplementary Fig. 1b). These findings demonstrate that prolonged exposure to high insulin and IGF1 increase the expression of senescence markers in human hepatocytes.

**Figure 2:**
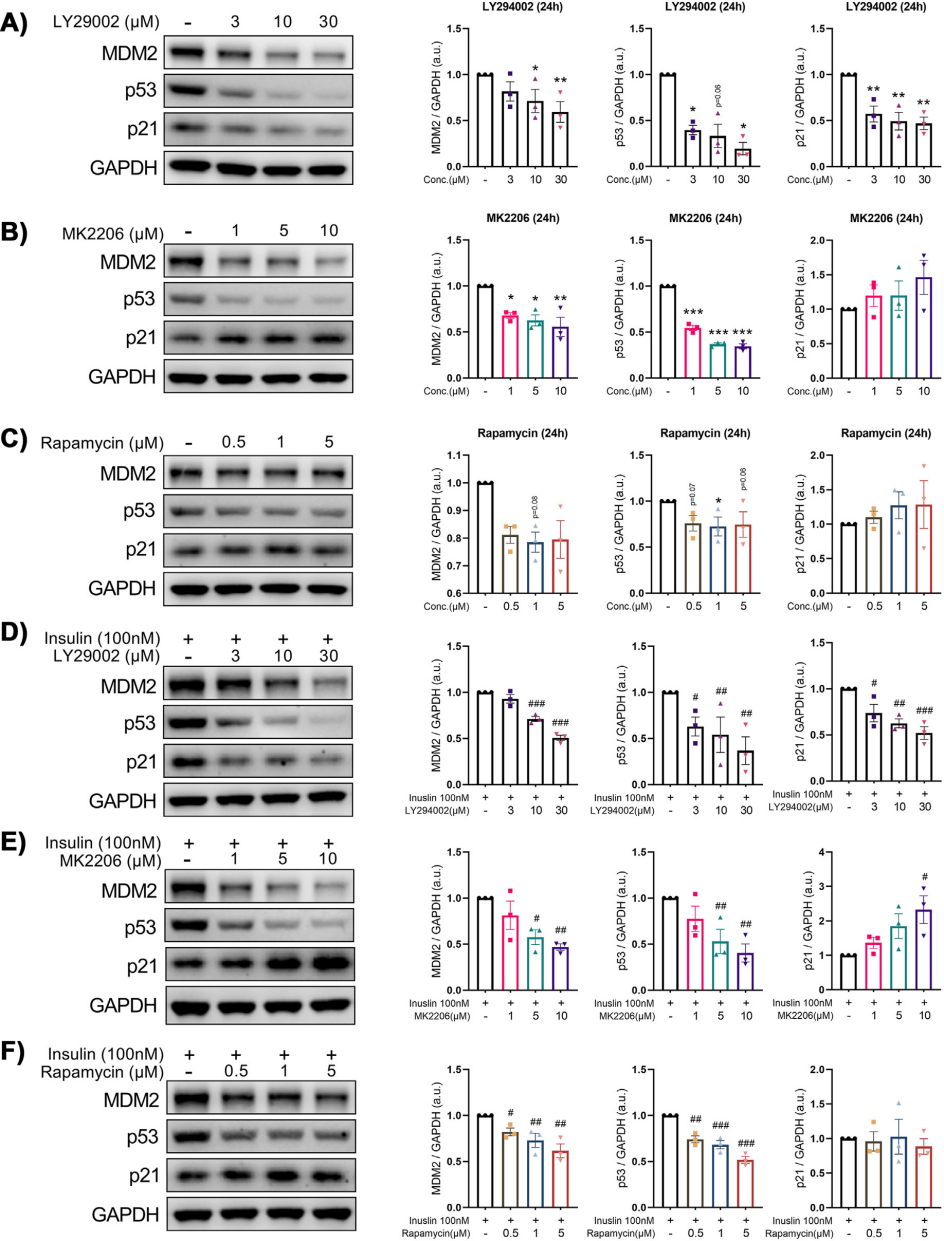
**Inhibition of the phosphoinositide 3-kinase (PI3K)/AKT/mammalian target of rapamycin (mTOR) pathway reduces insulin induction of senescence markers in human hepatocytes**. (A–C) Representative immunoblots of respective proteins in IHH cells treated for 24 h with the indicated concentrations of LY29002 (A), MK2206 (B) or Rapamycin (C). Bar graphs showing the relative protein quantification, normalized to GAPDH (n = 3). (D–F) Representative immunoblots of respective proteins in IHH cells treated for 1 h with the indicated concentrations of LY29002 (D), MK2206 (E) or Rapamycin (F) and stimulated with insulin (100 nM) for another 24 h. Bar graphs showing the relative protein quantification, normalized to GAPDH (n = 3). Data are shown as mean ± SEM. Dots represent individual level data. Statistical significance was determined using one-way ANOVA followed by Dunnett *post-hoc* test or Kruskal–Wallis with *post-hoc* Dunn's test. ∗^,#^p < 0.05, ∗∗^,##^p < 0.01, ∗∗∗^,###^p < 0.001. ∗*vs.* Ctrl (untreated cells); ^#^*vs.* cells treated with only insulin. a.u., arbitrary unit.

The PI3K/Akt/mTOR pathway is a key signaling pathway involved in regulating cell metabolism, growth, proliferation, apoptosis and autophagy and is thought to play a role in the regulation of cell senescence [14,15]. To test this possibility, we treated IHH cells with inhibitors of the PI3K, AKT and mTOR pathways (LY294002, MK-2206, and Rapamycin, respectively) in the presence or absence of insulin. As shown in Figure 2A, expression of MDM2, p53 and p21was reduced by LY294002 in a dose-dependent manner. Similarly, MK2206 dose-dependently reduced the MDM2 and p53 protein levels while no effect on p21 was observed (Figure 2B). On the other hand, rapamycin alone significantly reduced MDM2 levels but showed no effect on p53 and p21 levels (Figure 2C). Although only LY294002 was effective in reducing insulin induction of p21, we observed that the effect of MK2206 and rapamycin were similar to that of LY294002 in attenuating insulin induction of MDM2 and p53 (Figure 2D–F). Interestingly, the increase in p21 levels by MK2206 in insulin-treated cells suggested that insulin could also regulate p21 via p53-independent pathways (Figure 2E).

Together, these findings indicate that the PI3K/AKT/mTOR signaling pathway is required for insulin-induced induction of senescence markers in human hepatocytes. To further strengthen this, we analyzed the hepatic expression of senescence markers in the liver-specific insulin receptor knockout (LIRKO) mice. These mice exhibit dramatic insulin resistance with marked hyperinsulinemia due to deletion of the insulin receptor gene in hepatocytes [10]. When compared to the controls, there was no significant difference in liver expression of the senescence markers p21, γH2AX, and CCND1 in LIRKO mice as assessed by immunoblotting (Supplementary Fig. 2a). Similarly, there was no difference in *p21, SA-Gal*, or *p5*3 mRNA levels (Supplementary Fig. 2b). In contrast, the senescence markers p21, γH2AX, and CCND1 were increased in the epididymal white adipose tissue, with intact insulin signaling, from the same LIRKO mice (Supplementary Fig. 2c). Overall, these findings strongly support the concept that chronic hyperinsulinemia induces hepatic cell senescence by activating its signaling pathways, and that similar effects may occur in other tissues including the adipose.

### 3.2 Prolonged exposure to hyperinsulinemia aggravates DOX-induced senescence in human hepatocytes

**Figure 3:**
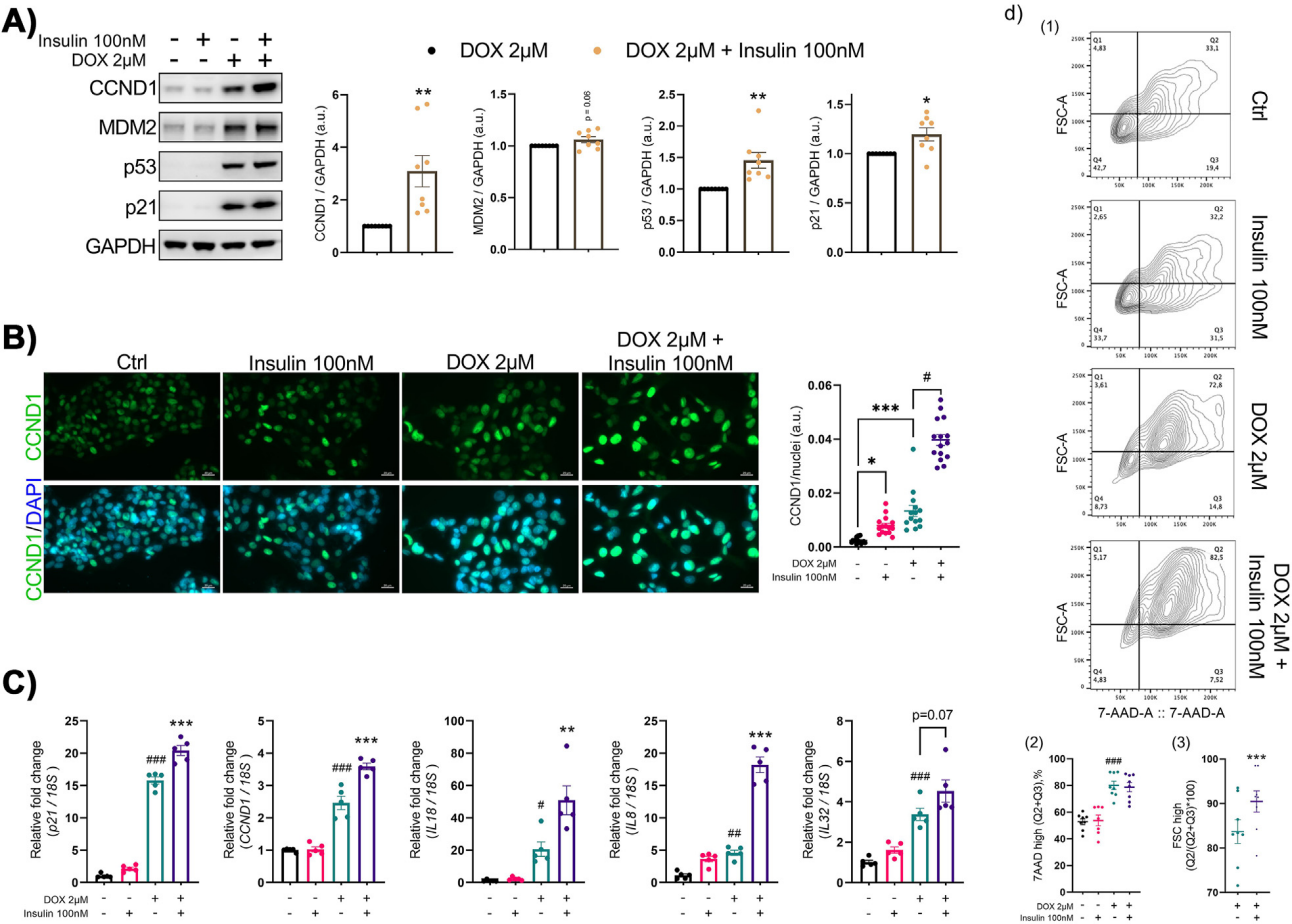
**Insulin worsens stress-induced senescence in human hepatocytes**. (A) Representative immunoblots of respective proteins in Ctrl and DOX-treated IHH cells, stimulated with or without insulin (100 nM) for 72 h. Bar graphs showing the relative protein quantification, normalized to GAPDH (n = 8). (B) Representative immunofluorescent images of Ctrl and DOX-treated IHH cells, stimulated with or without insulin (100 nM), stained for CCND1 (green) and nuclei (DAPI, blue). Scale bar represents 20 μm. A scatter plot displays fluorescence intensities quantified using ImageJ and normalized to number of nuclei (n = 3, 8–10 randomly chosen fields from each experiment). (C) RT-qPCR analysis of senescence markers (*p21, CCND1*) and SASP factors (*IL8*, *IL18* and *IL32*) in Ctrl and DOX-treated IHH cells, stimulated with or without insulin (n = 5). (D) Representative images of flow cytometry analysis by plotting forward scatter-area (FSC-A, cell size) versus 7AAD staining, in Ctrl and DOX-treated IHH cells, stimulated with or without insulin. The lower figure shows result quantitation. On the left, values are presented as percentage (%) of cells that are rich in DNA (Q2+Q3). On the right, values are presented as percentage (%) of the DNA rich cells with increased cell size. Data are shown as mean ± SEM. Dots represent individual level data. Statistical significance was determined by Student's t-test or one-way ANOVA followed by Bonferroni post-hoc test or Kruskal–Wallis with *post-hoc* Dunn's test. ∗^,#^p < 0.05, ∗∗^,##^p < 0.01, ∗∗∗^,###^p < 0.001. ∗*vs*. DOX-treated cells; ^#^*vs*. Ctrl cells. a.u., arbitrary unit.

We then evaluated whether hyperinsulinemia could render human hepatocytes more susceptible to stress-induced senescence, thereby accelerating liver senescence. To induce senescence in IHH cells, we used doxorubicin (DOX) which significantly increased the senescence markers MDM2, p53, and p21 (Figure 3A). Insulin exposure further enhanced the levels of these senescence markers in DOX-treated IHH cells (Figure 3A). Consistent with the findings in the mouse, hyperinsulinemia increased the mRNA levels of both *p21* as well as SASP factors *IL8*, *IL18*, and *IL32* in IHH cells (Figure 3C), demonstrating the pro-senescent effect of prolonged hyperinsulinemia also occurs in human liver cells.

Continuous exposure to hyperinsulinemia *in vivo* or *in vitro* induces cell cycle progression with a concomitant increase in DNA content, which triggers activation of the senescent cell program identified in mature adipocytes [8]. Cyclin D1 (*CCND1*), a downstream effector of the mitogenic insulin-signaling pathway, plays a key role in mediating the hyperinsulinemia-activated endoreplication and subsequent senescence in mature adipocytes [8]. Thus, we analyzed the expression of CCND1 in DOX-treated IHH cells in the presence and absence of insulin. Both mRNA and protein levels of CCND1 were significantly upregulated in DOX-induced senescent IHH cells (Figure 3A,C), which was further upregulated by insulin (Figure 3A,C). This insulin-inducing effect on CCND1 in DOX-treated cells was also confirmed by immunostaining (Figure 3B) and provide additional support to the notion that chronic hyperinsulinemia aggravates hepatocyte senescence acting through insulin-activated cell cycle dynamics. To further test this hypothesis, we analyzed DNA content in IHH cells after DOX and/or insulin treatment using flow cytometry. Staining of nuclear acids with 7-aminodactinomycin showed a significant increase in the frequency of cells with high DNA content after DOX or DOX + insulin treatment (Figure 3D), consistent with the acquisition of a senescent phenotype in hepatocytes that undergo gradual increase in DNA content [16]. As a result of increasing DNA content, there was a significant enlargement of the cell volume as assessed by the forward scatter in DOX + insulin treated IHH cells (Figure 3D), another conserved feature of senescence [17]. Thus, a combination of the increase in DNA content and in the cell volume in the presence of insulin implies a mechanism by which insulin can sustain cellular stress by increasing CCND1 expression and aggravating a senescent response.

We also tested this concept in our publicly available databases from patients with NAFLD/NASH [18]. Indeed, high *CCND1* mRNA levels in the livers from these patients associate with increasing expression of the senescence markers *p53*, *p21*, *p16* and *SA-βGal* as well as of the SASP factors *IL8* and *IL32* (Supplementary Fig. 3). Together, our findings provide a mechanistic and clinically sustainable link between hyperinsulinemia, liver senescence and the progression of NAFLD/NASH.

### 3.3 Co-treatment with senolytic agents dasatinib and quercetin counteracts the senescent expression program induced by insulin

**Figure 4:**
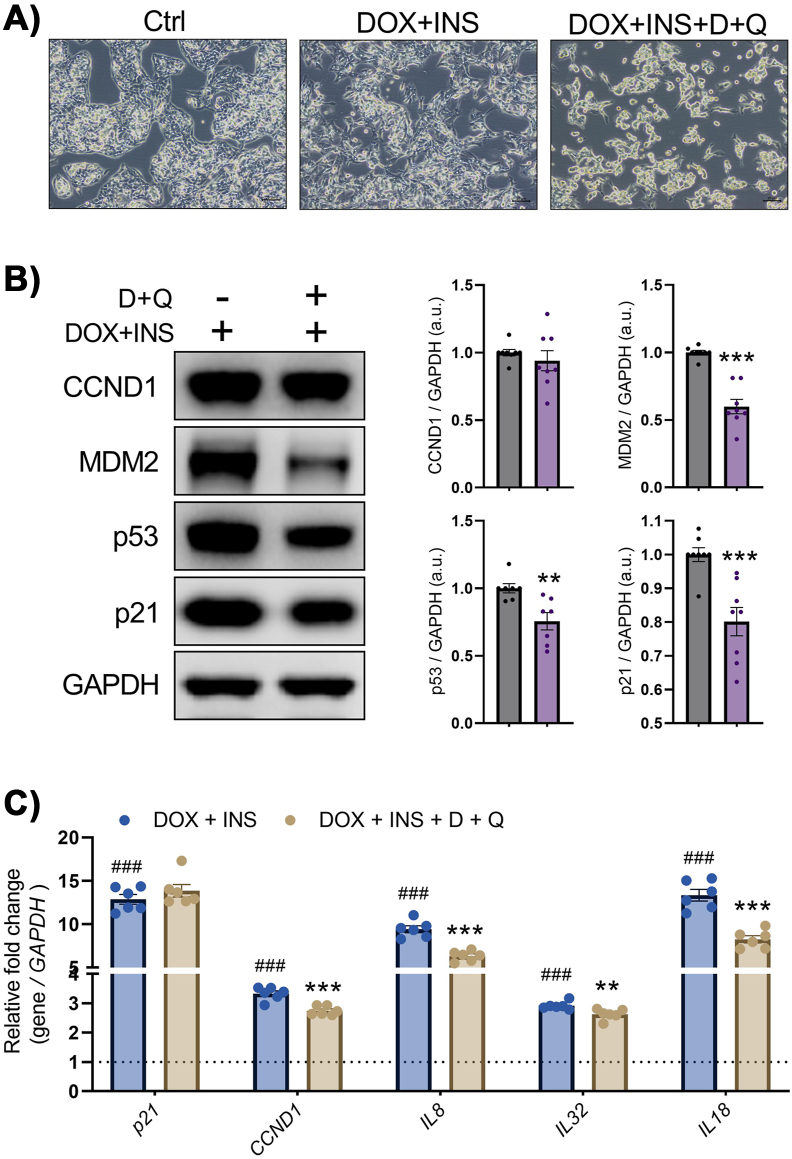
**Co-treatment with senolytic combination Dasatinib plus Quercetin (D + Q) counteracts the senescent expression program induced by insulin**. (A) Representative images (bright field) of IHH cells treated with DOX + Insulin or DOX + Insulin + D + Q. Scale bars represent 100 μm. (B) Representative immunoblots of respective proteins in DOX + Insulin-stimulated cells, treated with or without D + Q. Bar graphs showing the relative protein quantification, normalized to GAPDH (n = 7–8). (C) RT-qPCR analysis of senescence markers (*p21, CCND1*) and SASP factors (*IL8*, *IL18* and *IL32*) in DOX + Insulin-stimulated cells treated with or without D + Q (n = 6). Data are shown as mean ± SEM. Dots represent individual level data. Statistical significance was determined by Student's t-test or one-way ANOVA followed by Bonferroni *post-hoc* test. ∗p < 0.05, ∗∗p < 0.01, ∗∗∗^,###^p < 0.001. ∗*vs.* DOX + Insulin-treated cells; ^#^*vs.* Ctrl cells. a.u., arbitrary unit.

Since the elimination of senescent cells by treatment with a combination of the senolytic drugs dasatinib plus quercetin (D + Q) reduces overall hepatic steatosis in ageing, obese and diabetic mice [5], we tested the efficacy of this treatment in preventing insulin-induced hepatocyte senescence. IHH cells exposed to DOX + Insulin were cultured in the presence or absence of D + Q. The D + Q treatment reduced the number of cells, as shown in the bright field microscopic images (Figure 4A), consistent with ablation of senescent cells. Moreover, D + Q prevented the increase in protein levels of MDM2, p53 and p21 while no clear effect on CCND1 was observed (Figure 4B). However, the increase in mRNA levels of *CCND1*, *IL8*, *IL32*, and *IL18* was prevented by the combination of D + Q while no effect on *p2*1 mRNA was observed (Figure 4C). Thus, D + Q effectively inhibited induction of senescence in human hepatocytes by chronic hyperinsulinemia.

## 4 Discussion

Multiple risk factors including type 2 diabetes, obesity, and hyperinsulinemia predispose for the development and progression of NAFLD [1]. As people age, they become more vulnerable to these risk factors, which can be explained in part at the cellular level by the increased senescence [19]. Thus, cellular senescence represents a potential target of ageing- and metabolic-related diseases [20,21]. Growing evidence supports a role of cellular senescence of hepatocytes in the development of NAFLD and its progression towards NASH [22,23], and both obesity and T2D are associated with increased cell senescence [8,[24], [25], [26]]. However, the specific factors and mechanisms regulating hepatocyte senescence are still unknown. In a recent study, we found a strong association between increase in both fasting insulin levels as well as in degree of insulin resistance with hepatic senescence in NAFLD/NASH patients with obesity and T2D (our unpublished data). However, these are associative findings and no direct effects of hyperinsulinemia on senescence induction in human hepatocytes have been reported.

In the present work, we provide the first evidence that chronic hyperinsulinemia *in vitro* can initiate a senescence cell program in human hepatocytes via the p53/p21 signaling pathway. To further characterize the senescent phenotype induced by hyperinsulinemia, we stained IHH hepatocytes for γH2AX which, along with p53 and p21, is considered to be a robust and reliable marker for senescence [22]. IGF-I, a potent mitogenic factor with insulin-like activity [13], elicits a similar response in human hepatocytes, whereas treatment with insulin-signaling inhibitors (*i.e*., PI3K, AKT, or mTOR inhibitor) significantly reduces senescence markers, indicating that the PI3K/AKT/mTOR signaling pathway is required. Consistent with the *in vitro* findings, hyperinsulinemic LIRKO mice lacking insulin receptors in the liver showed no effect on senescence expression, whereas an increase in senescence was observed in the adipose tissue of these same mice where there is normal insulin receptor expression and intact insulin signaling. Together, these data show that insulin-dependent signaling pathways are critical for the induction of senescence by prolonged hyperinsulinemia. These findings are supported by a recent study showing that hyperinsulinemia can also induce senescence in mature adipocytes by activating mitogenic pathways downstream of the insulin receptor [8].

Senescence is a highly dynamic, multi-step process, during which the properties of senescent cells continuously evolve and can be influenced by several intrinsic and extrinsic insults such as telomere shortening, mitogenic signals, oncogenic signaling detection, oxidative and genotoxic stress, mitochondrial dysfunction or inflammation [4]. Thus, we wondered whether hyperinsulinemia could reinforce an existing senescence phenotype, potentially exacerbating senescence and accelerating ageing. For this, we used doxorubicin-treated IHH cells as an *in vitro* model and found that hyperinsulinemia further enhanced the senescence phenotype. Furthermore, insulin treatment resulted in a significant increase in Cyclin D1 levels as well as upregulation of senescence markers p53 and p21 and SASPs factors. Recent studies have shown that chronic hyperinsulinemia can induce cell cycle reentry and promote senescence in non-proliferating cells such as mature human adipocytes [8] or neurons [9]. Cyclin D1, which is known to initiate cell cycle entry, was found to be highly expressed in mature adipocytes isolated from hyperinsulinemic individuals as well as in neurons exposed to chronic insulin treatment [8,9]. We used flow cytometry to investigate the effect of hyperinsulinemia on endoreplication, *i.e.*, replication of the nuclear genome in the absence of mitosis leading to increased nuclear gene content and polyploidy. The size and DNA content of senescent cells exposed to hyperinsulinemia were significantly increased, indicating enhanced endoreplication and suggesting a mechanism by which insulin can sustain cellular stress by enhancing Cyclin D1 expression. Importantly, our analysis of a publicly available dataset [24] revealed a positive correlation between expression levels of Cyclin D1 and senescence markers in livers from NAFL/NASH patients, corroborating *in vitro* evidence of this study. This is also consistent with our recent study showing a strong association between insulin levels and hepatic senescence markers in NAFLD/NASH patients (our unpublished data). Overall, these findings support the link between hyperinsulinemia, Cyclin D1 and senescence and identify a mechanism by which hyperinsulinemic individuals can induce a senescent response.

Other studies have shown the therapeutic potential of senolytic drugs, like dasatinib and quercetin (DQ), in reducing the local burden of senescent cells in a variety of murine and human tissues [[27], [28], [29], [30]]. Our findings show that DQ antagonizes hyperinsulinemia-induced senescence in hepatocytes as well as reducing the expression of SASPs factors, implying that senolytics therapy may be beneficial for alleviating hyperinsulinemia-related liver senescence and associated complications.

As insulin resistance promotes hyperinsulinemia, there is an ongoing debate about whether senescence occurs before or after the onset of insulin resistance in T2D. Several studies have shown that chronic hyperinsulinemia lead to a state of selective insulin resistance, reducing signaling efficiency for certain insulin-signaling nodes while sparing others which could be responsible for inducing senescence [31,32]. Selective insulin resistance has also been observed in senescent human hepatocytes (*i.e.*, HepG2 cells) [33]. Furthermore, using LIRKO mouse as a model of pure insulin resistance in hepatocytes associated with hyperinsulinemia, we show that chronic insulin exposure requires the activation of insulin receptor signaling pathways to induce hepatic cellular senescence.

In conclusion, our findings show that prolonged hyperinsulinemia promotes hepatocyte senescence and that senolytics have an antagonistic effect. Our findings suggest that reducing insulin resistance and associated hyperinsulinemia can be an important strategy for halting senescence and disease progression in obese, type 2 diabetes and/or NAFLD individuals.

## Author contrbutions

R.K.B. and R.S. designed and performed experiments, analyzed data, and wrote the manuscript. M.C.E. performed flow cytometry analysis. BBB and ML provided the mice tissue samples. H.Y. performed the bioinformatics analysis. A.M., M.I.B., J.B., and C.R.K. critically reviewed the manuscript. U.S. conceived the study, critically reviewed the manuscript and edited it. All authors approved the final manuscript.

## Figures and Tables

**Figure:**
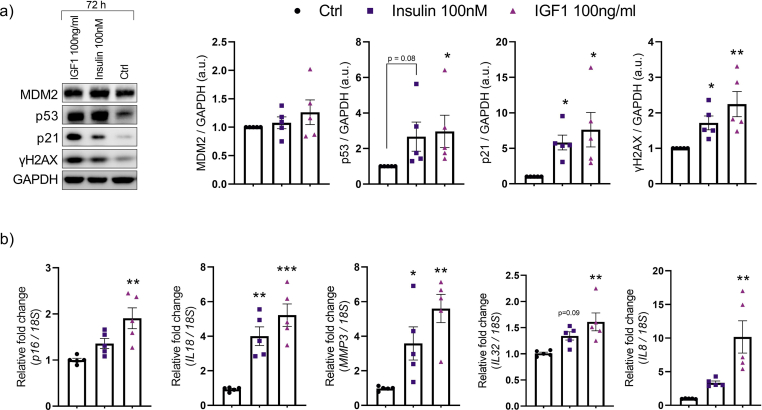
**Supplementary figure 1**: Effect on senescence markers in IHH cells following insulin or insulin-like growth factor 1 (IGF1) exposure for 72 h. (a) Representative immunoblots of respective proteins in IHH cells treated with insulin (100 nM) or IGF1 (100 ng/ml) for 72 h. Bar graphs showing the relative protein quantification, normalized to GAPDH (n = 5). (b) RT-qPCR analysis of senescence markers (*p16*) and SASP factors (*IL18, MMP3, IL32* and *IL8*) in IHH cells treated with insulin (100 nM) or IGF1 (100 ng/ml) for 72 h (n = 5). Data are shown as mean ± SEM. Dots represent individual level data. Statistical significance was determined using one-way ANOVA followed by Dunnett *post-hoc* test. ∗p < 0.05, ∗∗p < 0.01. ∗*vs.* Ctrl. a.u., arbitrary unit.

**Figure:**
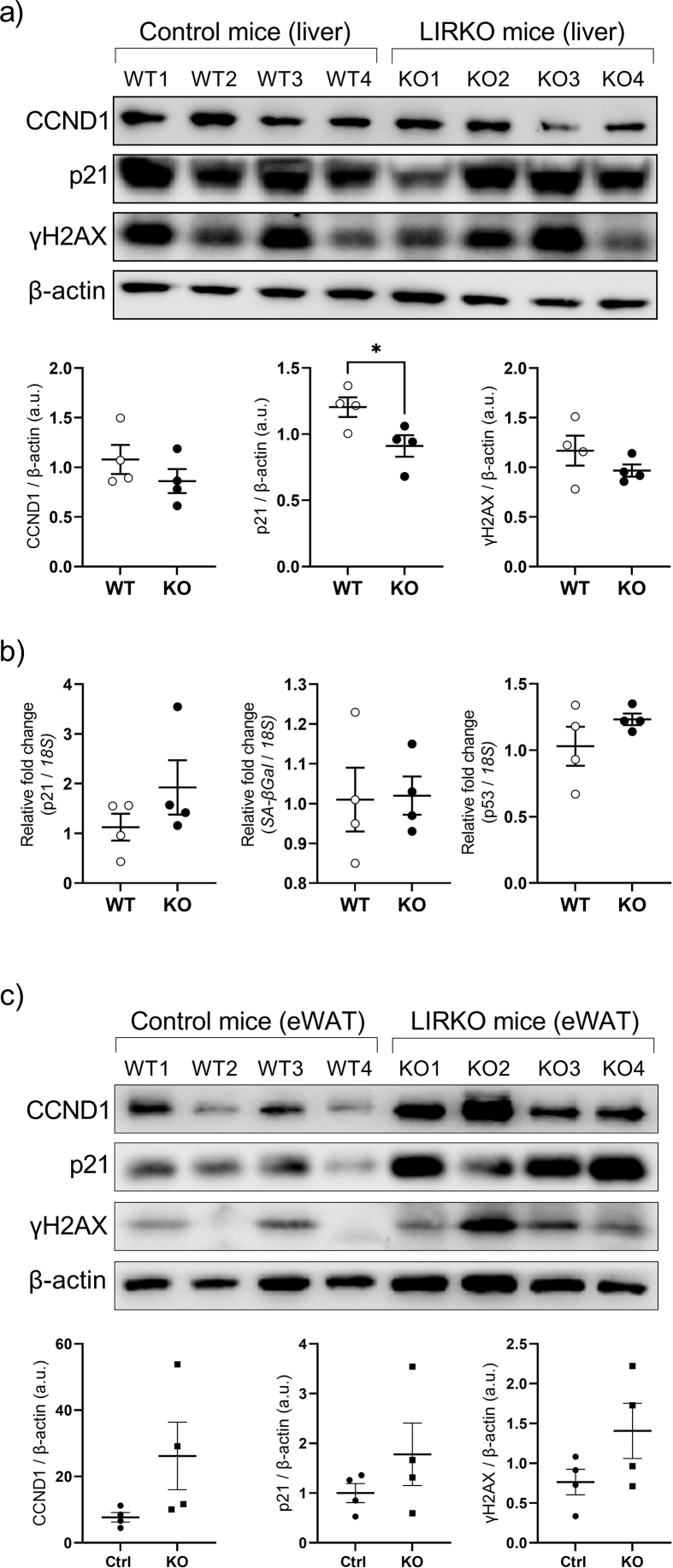
**Supplementary figure 2**: Expression of senescence markers in liver and epidydymal adipose tissue from LIRKO mice and their wild-type counterparts. (a-b) Representative immunoblots of respective proteins in the liver (a) and epidydymal adipose tissue (b) of wild-type and LIRKO mice. Bar graphs showing the relative protein quantification, normalized to β-actin (n = 4). (c) RT-qPCR analysis of senescence markers (*p21, p53, SA-βGal*) in livers of wild-type and LIRKO mice. Data are shown as mean ± SEM. Dots represent individual level data. Statistical significance was determined by Student's t-test ∗p < 0.05. ∗*vs.* Ctrl mice. a.u., arbitrary unit.

**Figure:**
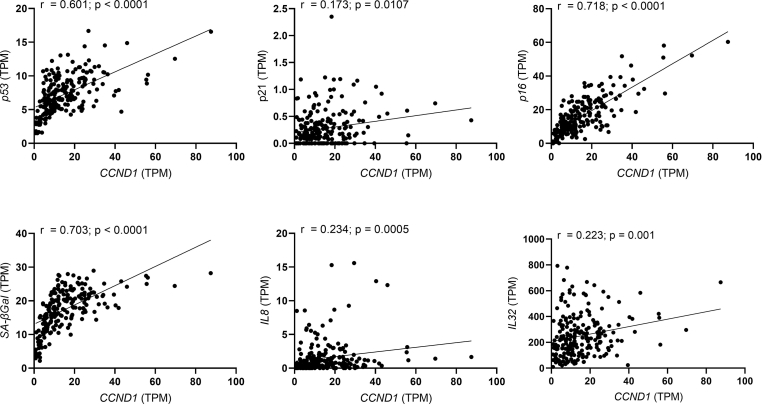
**Supplementary figure 3**: CCND1 is strongly associated with hepatic senescence markers in NAFL/NASH patients. Correlation analysis between mRNA levels of *CCND1* and senescence markers in the livers of healthy obese (n = 10), NAFL (n = 50) and NASH (n = 155) individuals (publically available dataset [18]). Spearman's correlation coefficient and p-value are indicated in the graph.

## Data Availability

Data will be made available on request.
