# Supplementary material for: Chronic hyperinsulinemia promotes human hepatocyte senescence
Source: Mol Metab. 2022 Jul 21;64:101558. doi: 10.1016/j.molmet.2022.101558 (PMC9364104; doi:10.1016/j.molmet.2022.101558)
Supplement: Multimedia component 1 [file mmc1.docx]

**Supplementary Table 1. Probes and primers used for quantitative real-time PCR.**

| Assays on Demands, ThermoFischer Scientific | |
| --- | --- |
| Gene | Assay number |
| *p21* | Hs00355782_m1 |
| *CCND1* | Hs00765553_m1 |
| *IL18* | Hs00155517_m1 |
| *IL32* | Hs00992441_m1 |
| *p21* | Mm00494449_m1 |
| *SA-βGal* | Mm00515342_m1 |
| *p53* | Mm01731287_m1 |
| In house design | |
| *IL8* | Forward GCGCCAACACAGAAATTATTGTA  Reverse TGAATTCTCAGCCCTCTTCAAA  Probe TTCTCCACAACCCTCTGCACCCAG |
| Endogenous Controls | |
| Euk 18S rRNA (20X) Thermo Fischer Scientific #4310893E (vic/TAMRA probe) | |
| Euk 18S rRNA (20X) Thermo Fischer Scientific #4319413E (vic/MGB probe) | |
